# Melatonin Supplementation Improves Cognitive Function in Type-2 Diabetes Mellitus: A Quasi-Experimental Study

**DOI:** 10.7759/cureus.95072

**Published:** 2025-10-21

**Authors:** Abida Pervaiz, Usman Pasha, Sadia Salman, Maham Zeba, Muhammad Zahid Jamil, Ismat Ullah

**Affiliations:** 1 Internal Medicine and Endocrinology, Jinnah Hospital Lahore/Allama Iqbal Medical College, Lahore, PAK; 2 Quality Control Labs, Department of Biochemistry, University of the Punjab, Lahore, PAK; 3 Paediatric Medicine, Manchester University NHS Foundation Trust, Manchester, GBR; 4 Medicine and Endocrinology, Sir Ganga Ram Hospital, Lahore, Lahore, PAK; 5 Medicine, Jinnah Hospital Lahore/Allama Iqbal Medical College, Lahore, PAK; 6 Endocrinology, Jinnah Hospital Lahore/Allama Iqbal Medical College, Lahore, PAK

**Keywords:** diabetes mellitus, effectiveness, impaired cognition, melatonin supplementation, moca

## Abstract

Introduction

Diabetes mellitus is a metabolic disorder characterized by dysregulation and alteration of glucose and lipid metabolism, leading to cognitive impairment. Impaired cognition is caused by a string of risk factors, among which glycemic dysregulation could be a reversible factor. The study aimed to evaluate the effectiveness of melatonin supplementation on impaired cognition in patients with type 2 diabetes mellitus, as assessed by changes in the Montreal Cognitive Assessment (MoCA) score.

Method

A quasi-experimental design (single-blinded) was conducted with a once-daily tablet of oral melatonin 5 mg at night for three months versus a placebo given in a cohort of 154 patients with type 2 diabetes mellitus after exclusion of a prior major neurological disorder. Patients having a mild to moderate cognitive impairment diagnosed via a validated tool, MoCA, with scores ranging from 10 to 25, were randomly assigned to two treatment groups (melatonin versus placebo). A follow-up at three months was carried out for the cognition scale and biochemical markers (HbA1C, low-density lipoprotein cholesterol (LDL-c), C-reactive protein (CRP), and serum uric acid), and compared with baseline parameters for both groups.

Results

A total of 154 subjects with a mean (SD) age of 49.98±10.08 years, a mean (SD) HbA1C of 9.49±1.50, and 90 (58.4%) female participants were allocated into two groups, i.e., Melatonin and Placebo. At 12 weeks of treatment, the melatonin-treated group showed improved MoCA score compared with the placebo group, mean (SD) MoCA, 21.35±4.75, OR=1.84, (95%CI, 0.355-3.33); p=0.016. The melatonin group also showed improvements compared to the placebo group with mean (SD) HbA1C, 8.68±1.38, OR=-0.585 (95%CI=-1.01-0.152); p=0.008, mean (SD) LDL-c, 154±34.10, OR= 1.01, (95%CI=-9.88-11.90); p=0.854, and mean (SD) CRP, 5.80±0.39, OR=-0.181 (95%CI=-0.305-0.057); p=0.004.

Conclusion

Melatonin supplementation in patients with type 2 diabetes mellitus led to significant improvement in cognitive function as assessed by the MoCA score, along with reductions in HbA1C and CRP levels. No significant effects were observed on LDL-c and uric acid. These findings suggest that melatonin may serve as a useful adjunctive therapy for improving cognition and certain metabolic parameters in diabetic patients.

## Introduction

Diabetes mellitus is a multi-system disorder characterized by glycemic and lipid dysregulation, leading to impaired quality of life by exerting its deleterious effects at the cellular level [1]. The prevalence of diabetes mellitus is substantially rising, with a huge burden on the health system, with an estimated 153 million people suffering from it, which will rise by 74% by 2045 [2]. Prompt diagnosis or screening is essential for developing countries to avoid expensive diabetes complications [3-5].

Management of diabetes mellitus, including lifestyle modification, pharmacological interventions to delay the progression of complications like neuropathy, nephropathy, liver disease, and cardiovascular disease by minimizing free radical oxidative stress and cellular injury [4]. Diabetes mellitus affects the neurological system, causing a constellation of features that range from a simple linguistic problem followed by dementia, speech deficit, verbal impairment, cognitive impairment, verbal thinking dysfunction, with diabetic encephalopathy being the most dreadful one [6].

Neurological complications in the form of vascular and non-vascular dementia, vascular occlusion, and neuropathy are one of the most important and untreated entities. Cognitive impairment may be progressive [7]. Cognition can be impaired due to various etiologies ranging from deficiency of micronutrients to genetic alteration. It has been estimated that cognitive impairment among diabetics ranges from 13.1% and 24.2% of those aged 65-74 and over 74 years, respectively. Primary care physicians will need to be proficient in recognizing and treating cognitive impairment as the number of older persons rises in the ensuing decades [8]. Cognitive decline and cognitive impairment have significant effects on patients and their families. Patients with mild cognitive impairment (MCI) benefit from aerobic exercise, mental stimulation, and cardiovascular risk factor management [7].

Melatonin is a crucial indolamine neuroendocrine hormone, synthesized from tryptophan and secreted by the pineal gland, and actively participates in controlling sleep patterns and cognitive abilities. Melatonin can efficiently regulate the immune system, has anti-stress properties, and regulates and alters the function of several organs [9]. Clinical research has shown that melatonin improves blood pressure, insulin metabolism, lipoprotein profiles, oxidative stress biomarkers, and inflammatory indicators in different ethnicities [10-12]. There is a strong association between cognitive impairment and low levels of serum melatonin in disorders associated with cognitive decline, like Alzheimer’s disease and the elderly with postoperative delirium [13]. Besides this, there is a potential association of melatonin and circadian regulation of insulin secretion by the pancreatic beta cells [14]. Serum melatonin levels is reduced significantly in both type 1 and type 2 patients, probably playing an important role in the genesis of diabetes [15]. Melatonin exerts anti-oxidative and immune-regulatory roles in people with diabetes [16].

Melatonin could have potentially beneficial effects, particularly in diabetes, and deficiency might influence the susceptibility to cognitive impairment [17,18]. The supplementation of melatonin is relatively safe among people, including children, women and adolescents, and young adults, in many illnesses like obstructive airway disease, epilepsy, and no major side effects were observed with improvement of sleep and preventing cognitive impairment in diabetes [19,20].

Given these converging lines of evidence, we conducted a single-center, quasi-experimental, single-blinded, placebo-controlled study aimed to evaluate whether 12 weeks of oral melatonin supplementation (5 mg nightly) improves cognitive function in adults with type 2 diabetes and mild-to-moderate cognitive impairment. Secondary objectives were to assess its effects on glycemic control (glycated hemoglobin (HbA1C)), lipid profile (low-density lipoprotein cholesterol (LDL-c)), uric acid, and systemic inflammation (C-reactive protein (CRP)) [21]. Neuronal dysregulation is a chronic process, so the study participants were enrolled with a disease duration of more than four years.

## Materials and methods

This was a quasi-experimental, single-blinded, placebo-controlled study conducted at the Jinnah Hospital Lahore, Lahore, Pakistan. The study was approved by the Ethical Review Board, Allama Iqbal Medical College, Jinnah Hospital, Lahore (approval number: ERB181/6/16-01-2025/S1), and conducted in strict accordance with the Declaration of Helsinki for research with human beings. The patients were recruited after a detailed examination, and informed consent was taken from each subject.

Study population

Inclusion criteria were patients of both genders having diabetes mellitus, no prior neurological disease in the form of stroke, and no history of vasculitis. Patients with a history of any autoimmune disease, or significant illness requiring hospitalization, hypertension (systolic and diastolic pressure above 150 mmHg and 100 mmHg, respectively), a refusal for consent, the use of fluvoxamine or any antioxidant supplement, working night shifts, smoking, drinking alcohol, breastfeeding, or being pregnant were not included. 

A total of 154 patients with type 2 diabetes with similar ethnicity and demographic backgrounds were finally included in the study. The participants were enrolled from the Diabetes and Endocrinology Department of Tertiary Care Hospital and were allocated equally into two groups by non-probability consecutive sampling. The Melatonin Group (n=77) was administered with melatonin capsules (5 mg/d) one hour before bedtime for 12 weeks, and the other group was given a placebo (Placebo Group). Both the melatonin and placebo capsules of the same shape and size were provided by the researchers, and patients were unaware of the contents of the package.

Data collection

**Figure 1:**
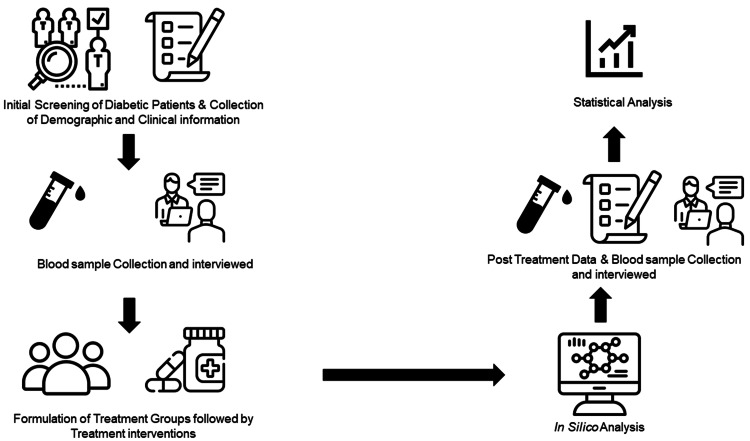
Work flow of study Image Credit: Dr Usman Pasha, author

The detailed clinical information collected at the time of recruitment included the medical and family history regarding disease duration, inheritance, and prevalence. The demographic information, including age, gender, education level, and disease duration for each patient, was also recorded along with the clinical information, such as the Montreal Cognitive Assessment (MoCA) score, biochemical markers (HbA1C, LDL-c, and uric acid), and immunological markers (CRP). Blood samples were taken for the baseline measurements of HbA1C, LDL-c, uric acid, and CRP. All the participants were assessed at the baseline and after 12 weeks of interventions by adopting a predefined implementation plan following standard operating procedure (Figure 1).

Outcomes

The primary outcome was global cognition assessed (MoCA scores) at 12 weeks. The MoCA was used to assess global cognition that ranged from 0 to 30, with higher scores indicating better cognitive function. Scores of 26-30 are considered normal, 18-25 are considered mild cognitive impairment, and 10-17 are considered moderate cognitive impairment [21]. The secondary outcomes were improvements in blood metabolic indices, including HbA1C, LDL, uric acid, and immunological markers like CRP. Search Tool for the Retrieval of Interacting Genes/Proteins (STRING) (https://version-9-1.string-db.org/), inBio Discover (ZS Associates, Evanston, Illinois, United States), and GeneMANIA Cytoscape plugin (University of Toronto, Canada) were used to predict the biological functions, gene-gene interactions, and co-expression of the receptor gene of melatonin [22].

Statistical analysis

IBM SPSS Statistics for Windows version 26 (IBM Corp., Armonk, New York, United States) was used for measuring the frequency and standard deviation of quantitative variables. Paired Student t-test analysis was performed for the evaluation of post-treatment effects, and one-way ANOVA was used to check the association between the MoCA score with demographic and clinical parameters of patients, such as age, disease duration, and CRP value. A p-value <0.05 and an odds ratio (OR) with a 95% confidence interval (CI) were considered statistically significant.

## Results

Demographical and clinical information

A total of 154 patients with diabetes were included in the study, and the baseline characteristics are given in Table 1. The mean age was 49.98±10.08 years, with the majority of patients having a mean disease duration of 6-10 years. The majority of patients were female (n=90; 58.4%).

**Table 1:** Demographic and clinical information of study subjects (N=154) Statistical analysis was performed using Paired student t-test* and Chi-Square Test ** (p<0.05 significant) BMI: body mass index; HbA1C: glycated hemoglobin; MoCA: Montreal Cognitive Assessment; SD: standard deviation; N/A: not applicable

Variable	Melatonin Group (n=77)	Placebo Group (n=77)	Total (n=154)	p- value
Age (years), mean±SD	50.49±11.73	49.46±8.16	49.98±10.08	0.530*
Gender, n (%)				0.252**
Male	28 (36.4%)	36 (46.8%)	64 (41.6%)	
Female	49 (63.6%)	41 (53.2%)	90 (58.4%)	
Disease Duration (years), n (%)				N/A
≤5	8 (10.40%)	13 (16.90%)	21 (13.7%)	
6-10	47 (61%)	36 (46.80%)	83 (53.8%)	
>10	22 (28.60%)	28 (36.30%)	50 (32.5%)	
Education, n (%)				0.892**
≤ Secondary School	69 (89.6%)	58 (75.32%)	127 (82.6%)	
Higher Secondary School	2 (2.6%)	08 (10.38%)	10 (6.60%)	
Graduation	2 (2.6%)	06 (7.80%)	08 (5.20%)	
Masters and above	4 (5.20%)	05 (6.50%)	09 (5.60%)	
BMI (kg/m^2^), n (%)				N/A
≤ 25	37 (48%)	29 (37.7%)	66 (42.9%)	
>25	40 (52%)	48 (62.3%)	88 (57.1%)	
HBA1C (%), mean ±SD	9.62±1.59	9.35±1.40	9.49±1.50	0.264*
MoCA score, mean ±SD	18.10±5.29	19.37±4.62	18.37±4.99	0.115*

The Melatonin Group had a low MoCA score and CRP of 18.10±5.29 and 5.82±0.344, respectively, and an elevated HbA1C of 9.62±1.59, high LDL-c of 165.68±36.68, compared to the Placebo Group (Table 2). The melatonin group showed significant improvement in MoCA score (OR= 1.84, 95%CI= 0.355-3.33; p=0.016), decreased HbA1C (OR= -0.585, 95%CI= -1.01-0.152; p=0.008) and CRP (OR= -0.181, 95% CI= -0.305-0.0577; p=0.004) compared to the Placebo Group after 12 weeks. Other variables, including LDL-c and uric acid, did not show statistically significant variation after treatment from baseline (OR= 1.01, 95%CI= -9.88-11.90; p=0.84) and (OR= 0.003, 95%CI= -0.340-0.358; p=0.983), respectively.

**Table 2:** Effect of melatonin and placebo administration on metabolic status in patients with diabetic mellitus (N=154) MoCA: Montreal Cognitive Assessment; HbA1C: Hemoglobin A1C; LDL (c): LDL-cholesterol; CRP: C-reactive protein; SD: Standard Deviation Statistical analysis was performed using Paired student t-test (p<0.05 significant)

Sr. No	Variable	Melatonin Treated Group	Difference (Δ)	p-value	Placebo Group	Difference (Δ)	p-value	Overall	Difference (Δ)	p- value	95%CI
1	MoCA score Before ±SD	18.10±5.29	4.17	0.001	19.37±4.62	1.05	0.12	18.37±4.99	2.98	0.115	-1.2 (-2.85-0.312)
2	MoCA score After ±SD	22.27±4.97			20.42±4.35			21.35±4.75		0.016	1.84 (0.355-3.33)
3	HbA1C Before % ±SD	9.62±1.59	1.24	0.001	9.35±1.40	0.45	0.001	9.49±1.50	0.81	0.264	0.271 (-0.20-0.74)
4	HbA1C After % ±SD	8.38±1.34			8.9±1.37			8.68±1.38		0.008	-0.585 (-1.01-0.152)
5	LDL (c) Before mg/dl ±SD	165.68±36.68	10.5	0.15	162±32.78	7.89	0.10	164±34.71	10	0.562	3.25 (-7.81-14.33)
6	LDL (c) After mg/dl ±SD	155.18±34.33			154.11±34.09			154±34.10		0.854	1.01 (-9.88-11.90)
7	Uric Acid Before mg/dl ±SD	7.57±1.04	0.18	0.23	7.72±1.22	0.33	0.31	7.65±1.13	0.26	0.405	-0.15 (-0.15-0.209)
8	Uric Acid After mg/dl ±SD	7.39±0.95			7.39±1.2			7.39±1.1		0.983	0.003 (-0.340-0.358)
9	CRP Before IU/mL ±SD	5.82±0.344	0.11	0.001	6.05±0.53	0.16	0.001	5.94±0.45	0.14	0.002	-0.223 (-0.365-0.081)
10	CRP After IU/mL ±SD	5.71±0.339			5.89±0.43			5.80±0.39		0.004	-0.181 (-0.305-0.0577)

In silico analysis of selected gene

**Figure 2:**
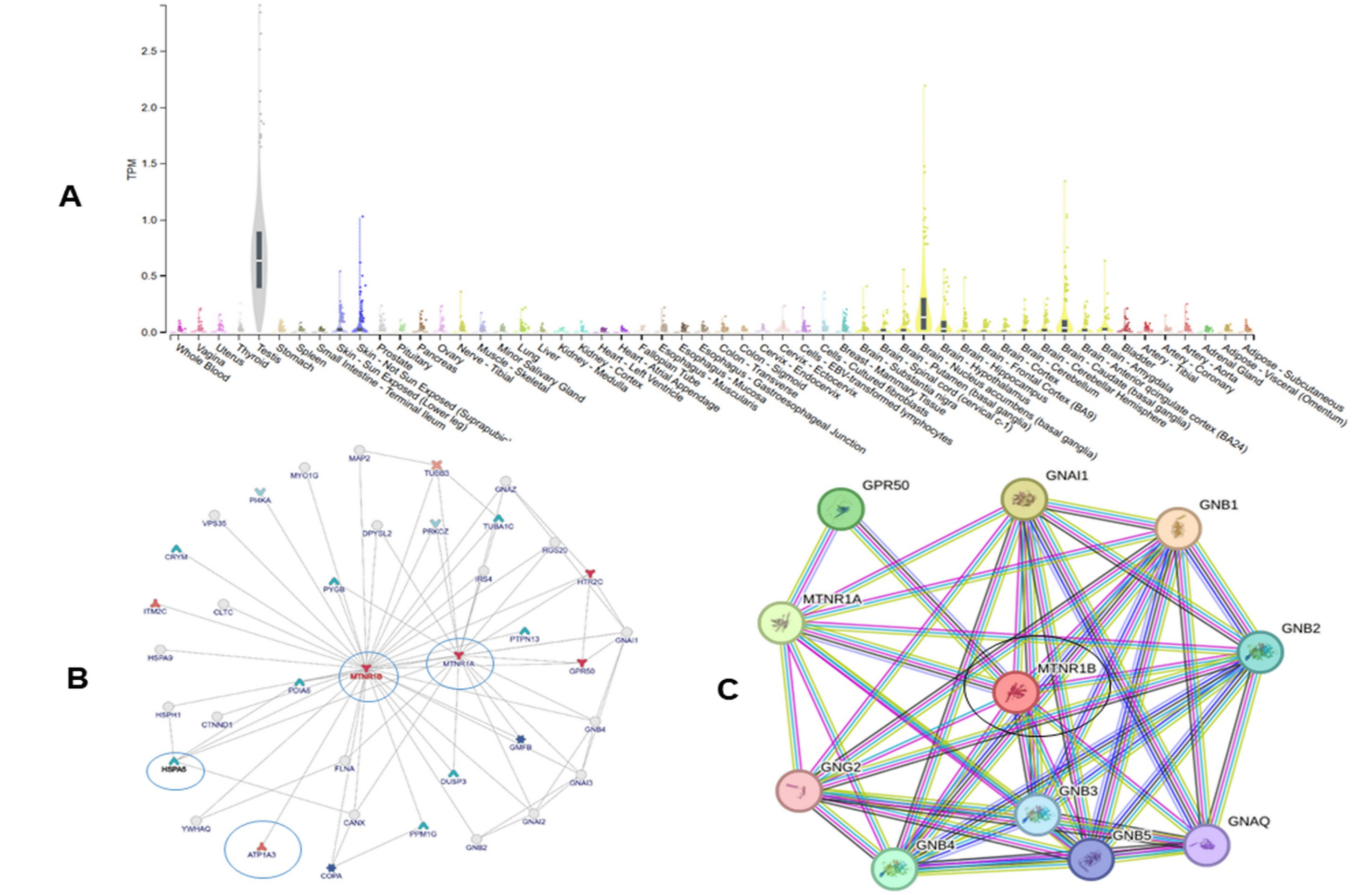
(A) Violin plot represent the abundance of melatonin receptor in different tissues; (B) Interactive pattern of melatonin receptor gene (MTNR1B) using inBio Discover; (C) Interactive pattern of melatonin receptor gene (MTNR1B) using STRING software STRING: Search Tool for the Retrieval of Interacting Genes/Proteins

The expression, gene regulation, and interaction of the melatonin binding receptor gene were assessed using different in silico tools (Figure 2A). The highest expression was found in testis cells, while cells associated with the brain and memory, such as the caudate, nucleus accumbens, amygdala, etc., have comparatively high expression of the receptor gene. Results from inBio Discover indicated that the interaction of melatonin receptor (MTNR1B) with MTNR1A, PD1A6, HSPA5, COPA, and ITM2C, etc., all play a key role in modulating memory (Figure 2B). Data obtained from STRING also confirmed the role of MTNR1A as a key regulator of brain function (Figure 2C).

## Discussion

We carried out a quasi-experimental study to evaluate the impact of melatonin and its association with improvement in cognitive function (MoCA score), along with glycemic profile (HbA1C). Our main findings were as follows: (i) MoCA score was significantly improved in the melatonin-treated group than the placebo group, (ii) HbA1C and CRP were improved significantly (p<0.05) with melatonin treatment, (iii) LDL-c and uric acid were found to be insignificantly related to melatonin treatment. These findings suggest that melatonin administration was associated with improved cognitive performance and better glycaemic control (as reflected by the reduction in HbA1C), with concomitant decreases in systemic inflammation (CRP).

In the previous studies, it has been reported that cognitive impairment is a poorly addressed domain in the management of diabetes, with continuous deterioration as diabetes progresses. Additionally, it was established that disease-associated alterations in cortical thickness and white matter integrity were more obvious with obesity and raised baseline CRP levels than with patients of normal weight with diabetes. These outcomes indicate that weight status and baseline raised inflammatory markers may play additive roles in neuronal dysfunction associated with diabetes.

Melatonin is an anti-aging protein that helps with aging-related changes, such as cognitive decline. Moreover, it is associated with aging, with a reported steady decrease in concentration with aging [23]. The current study showed that melatonin supplementation was associated with improvement of cognition in early stages of neuronal dysregulation. In the current study, the MoCA score was observed as 18.37±4.99, which is similar to the MoCA score reported in a previous study in a similar ethnicity [24]. Our single-blinded, placebo-controlled trial in adults with type 2 diabetes having mild-moderate cognitive impairment (assessed as MoCA score) treated with 12 weeks of nocturnal oral melatonin (5 mg) revealed marked improvement in cognition (MoCA 22.27±4.97; p=0.001) with a modest yet significant improvement in glycemic control (HbA1C 8.38±1.34: Δ= -1.24, p=0.001). Increased recognition of the risks associated with neurological dysfunction is essential for the prevention and management of brain atrophy and cognitive dysfunction connected to type 2 diabetes from its very early stages. Various studies have reported the effectiveness of melatonin for cognitive functions in various diseases, including diabetes [25]. Melatonin also improves sleep quality and cognitive functions by improving the MoCA score in patients undergoing hemodialysis [25].

We have also investigated the impact of melatonin on CRP levels, which were significantly improved in the melatonin-administered group in our study. Chronic inflammatory reactions are linked to the pathogenic processes of a number of diseases, including diabetes. Actually, hyperglycemia can cause diabetes-related problems, including diabetic neuropathy and cognitive impairment by activating NFκB, a key inflammatory signal pathway [26]. Pro-inflammatory cytokine production and pathological brain inflammation are linked to NFκB. Patients with type 2 diabetes have higher levels of several pro-inflammatory markers, such as mean platelet volume, uric acid/HDL (high-density lipoprotein) ratio, neutrophil/lymphocyte ratio, platelet/lymphocyte ratio, and CRPs [27-29].

Our findings augment those of prior research, which relates the biology of melatonin to neurocognitive and metabolic circuits associated with diabetes. Mechanistically, melatonin has antioxidant qualities, reduces low-grade inflammation, and modifies the circadian regulation of pancreatic β-cell function and glucose homeostasis processes linked to diabetes-related brain damage and cognitive decline [9,14,16,26]. NF-κB-mediated signaling plays a pivotal role in diabetes-associated neuro-inflammation, and our observed decrease in CRP is directionally consistent with melatonin's immune-regulatory effects [26-28]. Our findings using bioinformatics tools is strengthened by the results of the previous studies where the significant improvement in mitochondrial function with decreased apoptosis (often working in concert with NAD+ precursors) in the cortex and hippocampus was observed with melatonin supplementation and decreased circulating melatonin in patient groups at risk for postoperative delirium and MCI [13,18].

Perhaps due to (i) the shorter exposure duration compared to the slower kinetics of lipid remodeling, (ii) background statin or urate-active therapies that may have decreased detectable between-group deltas, and/or (iii) limited power for minor metabolic changes, there was no effect on LDL-c or uric acid over the 12-week period of treatment. The main clinical benefit of melatonin may be through glycemic/inflammatory axes rather than lipid regulation during the testing period, as evidenced by the constant improvement of HbA1C and CRP, two integrative biomarkers with comparatively rapid reactivity [29]. A ~1.8-point group difference in MoCA is small from a clinical perspective, but it may have significance at the cohort level, especially for individuals whose cognitive condition may affect how they manage their diabetes [7, 8].

A mechanistic association between melatonin signaling and cognitive domains is supported by our exploratory in silico analyses, which highlight biological plausibility. Robust expression of melatonin receptor genes in memory-related brain regions and network connectivity of MTNR1A/MTNR1B with proteins involved in synaptic function and proteostasis [22]. Even while these bioinformatics results provide predictions, they support the clinical signal and offer particular targets (such as MTNR1B) for prospective pharmacogenetic or receptor-specific research.

Clinically, melatonin has been shown to have a good tolerability profile in the literature; our data suggest the use of melatonin as an adjuvant for patients with type 2 diabetes and mild-moderate cognitive impairment, particularly when sleep/circadian disturbance and inflammatory activation are present [9-11,16,17]. Nevertheless, with consideration for concurrent diabetic, antidepressant, and sedative medications, therapy needs to be tailored and well monitored. Future research priorities include domain-specific cognitive testing, longer follow-up to assess durability and relapse, expanded inflammatory/oxidative biomarker panels to resolve mediators and moderators of benefit, and dose-finding and timing (chronotherapy) [1,18,23].

Convergence across the clinical and molecular domains, pre-specified endpoints encompassing inflammation, glycemia, and cognition, and randomization vs placebo strengthen our study. The single-center study design, single melatonin dosage, shorter duration, lack of complete sleep/circadian phenotyping, and the use of a global screen (MoCA) in place of an extensive neuropsychological battery are some limitations of our study. Additionally, we did not measure levels of metabolites or circulating melatonin, which would have precluded exposure-response analysis. Finally, future multi-center trials powered for important secondary endpoints are necessary even though attrition was minimal and allocation was balanced (n=77 per arm at randomization).

## Conclusions

This single-center quasi-experimental study demonstrated that 12 weeks of nightly melatonin supplementation (5 mg) significantly improved cognitive performance and reduced HbA1C and CRP levels in patients with type 2 diabetes and mild to moderate cognitive impairment. No significant changes were observed in LDL-c or uric acid levels. These findings suggest that melatonin is a safe, well-tolerated, and cost-effective adjunct therapy with potential benefits for both cognitive and metabolic control in diabetic populations. Despite growing evidence linking diabetes-related metabolic dysregulation to cognitive decline, the role of melatonin in modulating this relationship remains underexplored - particularly in clinical populations with established diabetes.

This study helps to address this research gap by providing preliminary clinical evidence supporting melatonin’s beneficial effects on both cognitive and biochemical parameters. Future large-scale, multi-center randomized controlled trials with longer follow-up, detailed neuropsychological testing, and comprehensive biomarker profiling are needed to validate these results and clarify the underlying neuroprotective mechanisms of melatonin in diabetes.
